# Book Review of Surface Guided Radiation Therapy edited by Jeremy D. P. Hoisak, Adam B. Paxton, Benjamin Waghorn and Todd Pawlicki

**DOI:** 10.1002/acm2.13082

**Published:** 2020-11-12

**Authors:** Wolfgang A. Tomé

**Affiliations:** ^1^ Department of Radiation Oncology Division of Therapeutic Medical Physics Montefiore Medical Center and Albert Einstein College of Medicine Bronx NY USA

**Keywords:** SGRT

1

This monograph on surface guided radiation therapy (SGRT) covers all of the essential aspects of surface image guidance applied to radiation therapy, beginning with a historical overview and followed by a very nice chapter on safety and quality improvement. Following these two introductory chapters, the monograph can then be divided into four main sections: (i) the technical overview of currently available commercial SGRT Systems (chapters 3–5), (ii) the commissioning of the systems described in part (i) (chapters 6–8), (iii) the clinical application of SGRT (chapters 9–19), and (iv) miscellaneous topics in surface image guidance (chapters 20–26). While the corresponding chapters in part (i) and (ii) can be read independently of each other, I personally would have found it more helpful if the technical overview of a particular surface image guidance technology would have been followed by the chapter on its commissioning. Having said that, each of the chapters in part (i) and (ii) strikes the right balance between technical detail and brevity, making them very useful both as a reference and an introduction to the technology and how to commission a given technology of surface image guidance. Part (iii) begins with an in depth look at the clinical application of this technology to breast cancer (chapters 9–11), while Chapters 12–14 consider Stereotactic Radiosurgery and Stereotactic Radiation Therapy in depth, including a very nice chapter on the risk analysis for SGRT‐based SRS/SRT. Part (iii) continues with a detailed description of respiratory motion management strategies employing surface image guidance in SBRT (chapter 15), including the use of surface image guidance for SBRT without respiratory gating (chapter 16). Chapters on the use of surface image guidance in the treatment of Head and Neck (chapter 17), extremities (chapter 18), and pediatric patients (chapter 19) conclude Part (iii). Chapters 20–26 round out the presentation with three perspectives on the clinical implementation of surface image guidance, one from the point of view of a physicist and two from the that of a radiation therapist (chapters 20–22); applications of surface image guidance to proton therapy (chapter 23); integration of surface image guidance into tomographic and bore type gantry systems (chapter 24); use of consumer grade cameras and alternate approaches to surface image guidance (chapter 25); and a final chapter discussing future directions for SGRT (chapter 26).

In summary, this monograph presents a very readable account of the technical features of currently commercially available SGRT technologies, considers the commissioning of each in detail, and discusses in detail the clinical implementation and use of these systems for a number of important clinical sites, such as Breast, Stereotactic Body Radiation Therapy, Stereotactic Radiosurgery and Stereotactic Radiation Therapy, and Head and Neck. Regardless of career stage, professionals in either therapeutic medical physics or radiation oncology will find this monograph beneficial. The authors of this monograph balance technical detail with clinical information regarding the technology's implementation, and, hence, those interested in SGRT will find this monograph a valuable and welcome addition to their professional library. I have enjoyed reading through this monograph and found it a cogent reference and a thorough introduction to the topic.

Wolfgang A. Tomé, Ph.D., FAAPM, FASTRO, is a Professor of Radiation Oncology and a Professor of Neurology at Albert Einstein College of Medicine (Bronx, New York) and the Chief of the Division of Therapeutic Medical Physics at Montefiore Medical Center (Bronx, New York).

